# Assessment of Glial Scar, Tissue Sparing, Behavioral Recovery and Axonal Regeneration following Acute Transplantation of Genetically Modified Human Umbilical Cord Blood Cells in a Rat Model of Spinal Cord Contusion

**DOI:** 10.1371/journal.pone.0151745

**Published:** 2016-03-22

**Authors:** Yana O. Mukhamedshina, Ekaterina E. Garanina, Galina A. Masgutova, Luisa R. Galieva, Elvira R. Sanatova, Yurii A. Chelyshev, Albert A. Rizvanov

**Affiliations:** 1 Institute of Fundamental Medicine and Biology, Kazan (Volga Region) Federal University, Tatarstan, Kazan, Russia; 2 Department of histology, Kazan State Medical University, Tatarstan, Kazan, Russia; University of Toronto, CANADA

## Abstract

**Objective and Methods:**

This study investigated the potential for protective effects of human umbilical cord blood mononuclear cells (UCB-MCs) genetically modified with the *VEGF* and *GNDF* genes on contusion spinal cord injury (SCI) in rats. An adenoviral vector was constructed for targeted delivery of VEGF and GDNF to UCB-MCs. Using a rat contusion SCI model we examined the efficacy of the construct on tissue sparing, glial scar severity, the extent of axonal regeneration, recovery of motor function, and analyzed the expression of the recombinant genes *VEGF* and *GNDF in vitro* and *in vivo*.

**Results:**

Transplantation of UCB-MCs transduced with adenoviral vectors expressing VEGF and GDNF at the site of SCI induced tissue sparing, behavioral recovery and axonal regeneration comparing to the other constructs tested. The adenovirus encoding VEGF and GDNF for transduction of UCB-MCs was shown to be an effective and stable vehicle for these cells *in vivo* following the transplantation into the contused spinal cord.

**Conclusion:**

Our results show that a gene delivery using UCB-MCs-expressing *VEGF* and *GNDF* genes improved both structural and functional parameters after SCI. Further histological and behavioral studies, especially at later time points, in animals with SCI after transplantation of genetically modified UCB-MCs (overexpressing VEGF and GDNF genes) will provide additional insight into therapeutic potential of such cells.

## Introduction

Numerous experimental studies have found that transplantation of genetically modified cells carrying a transgene has a greater stimulating effect on the regeneration of post-traumatic central nervous system [1,2,3,4]. During spinal cord injury (SCI), the extensive area adjacent to the epicenter of the injury gets involved in the pathological process. As such, in order to achieve complete therapeutic action, the therapeutic gene must be delivered not only to the epicenter of traumatic injury, but also to the surrounding areas distant from the epicenter of injury. To solve this problem, cellular carriers are used as an adequate instrument for the delivery of therapeutic genes. Cells intended for transplantation and for use as therapeutic gene carriers may themselves act as targets for the transgene gene products [5]. This can promote their survival, migration potential, and control the expression of the phenotypic characteristics.

In this study, we chose human umbilical cord blood mononuclear cells (UCB-MCs), which are easy to produce [6,7] and have been demonstrated to be safe, have low immunogenicity, as well as having the potential for increasing neuroregeneration [8,9]. Previously, we have shown that transplantation of UCB-MCs, transduced with a recombinant adenoviral vector encoding glial cell-derived neurotrophic factor (GDNF), into the injured spinal cord of rats contributed to the restoration of motor function and improved tissue sparing [10]. Further, transplantation of UCB-MCs expressing vascular endothelial growth factor (VEGF) increased capillary density in the ischemic area, improved blood flow and promoted the formation of new blood vessels [11]. VEGF and GDNF are powerful factors in the maintenance of viability of a number of cell different populations in the spinal cord, including the motor neurons [12,13]. VEGF stimulates neurogenesis and axonal growth [14] as well as the proliferation of astrocytes [15], neural stem [16], and Schwann [17] cells. GDNF reduces apoptosis and tissue degeneration [18], supports expression of neurofilament protein, calcitonin gene related peptide (CGRP) and growth associated protein 43 (GAP-43) [12]. Considering the action of VEGF and GDNF through different receptors and pathways, it is reasonable to hypothesize that the simultaneous delivery of these two therapeutic genes promotes synergistic neuroprotective effects. Previously, we engineered UCB-MCs transduced with adenoviral vectors encoding VEGF and GDNF for the treatment of amyotrophic lateral sclerosis (ALS) and demonstrated a prominent symptomatic control and prolonged life-time in ALS mice after transplantation [19]. Genetically modified VEGF and GDNF UCB-MCs facilitated targeted delivery of the recombinant therapeutic molecules to motor neurons and extended the survival of the neurons.

In this paper, we explore the use of this construction as a stimulant of neuroregeneration after SCI. We hypothesized that UCB-MCs genetically modified with recombinant adenoviral vectors encoding VEGF and GDNF would have various effects on the processes of neuroregeneration after SCI. To examine the efficacy of this construct we used the rat contusion SCI model, to evaluate tissue sparing, glial scar severity, the extent of axonal regeneration, recovery of motor function, in addition to analysis of the expression of VEGF and GDNF.

## Materials and Methods

### Isolation and adenoviral transduction of UCB-MCs

Umbilical cord blood was obtained from healthy full-term pregnant women with gestational age 39–40 weeks with in accordance with the Protocol and Standards of the Stem Cell Bank of Kazan State Medical University. The study was approved by the Institutional Review Board of Kazan State Medical University. Written informed consent was obtained from each subject according to the clinical and experimental research protocol, approved by the Local Ethic Expert Committee of the Kazan State Medical University (number 195, 10 May 2010). Isolation of mononuclear blood cells and generation of adenoviral vectors was performed as described previously [10]. After purification, UCB-MCs were maintained in RPMI-1640 medium (Sigma, USA) supplemented with 10% FBS (Sigma), penicillin and streptomycin (100 U/ml, 100 μg/ml, respectively, Sigma). Immediately after isolation, UCB-MCs were seeded in 10 cm culture dishes and transduced with the recombinant adenoviruses Ad5-VEGF and Ad5-GDNF, or Ad5-EGFP (enhanced green-fluorescent protein) at an MOI of 10. Cells were incubated for 12–16 h in a humidified atmosphere of 5% CO_2_ at 37°C. Prior to injection, the cells were washed with Dulbecco`s Phosphate Buffered Saline (DPBS, Paneco, Russia).

### Animals and experimental groups

Forty three adult male and female Wistar rats (weight, 250–300 g each; Pushchino Laboratory, Russia) were randomly assigned to four groups. UCB-MCs, 2×10^6^ cells per rat, were injected after SCI or laminectomy. The groups were: (1) UCB-MCs transduced with recombinant adenoviral vectors encoding VEGF and GDNF (SCI UCB-MCs+Ad5-VEGF+Ad5-GDNF, n = 12). (2) UCB-MCs transduced with recombinant adenoviral vector encoding therapeutically inactive EGFP (SCI UCB-MCs+Ad5-EGFP, n = 10), (3) no injections (SCI, n = 11), and (4) after laminectomy without SCI, injection of UCB-MCs+Ad5-VEGF+Ad5-GDNF (Sham UCB-MCs+Ad5-VEGF+Ad5-GDNF, n = 10). All animal procedures and care were approved by the Kazan State Medical University Animal Care and Use Committee (Permit Number: 5 dated 27 May 2014), and experimental protocols were consistent with the recommendations of the Physiological Section of the Russian National Committee on Bioethics. Animals were group housed in clear plastic cages (12 h:12 h light/dark cycle) with food and water available ad-libitum.

### Spinal cord injury

Rats were deeply anesthetized by intraperitoneal injection of chloral hydrate (80 mg/ml, 0.4 ml per 100 g, Sigma). After skin incision, laminectomy at the Th8 vertebral level was performed. The impact rod (diameter 2 mm, weight 10 g) of an impactor was centered above Th8 and dropped from a height of 25 mm to induce SCI [20]. Immediately after laminectomy or SCI, in animals receiving injections, UCB-MCs were injected intraspinally into two points (5 μL/injection; 1 × 10^6^ cells/5 μL DPBS). The distance from each injection point of to the center of the injury was 1 mm. The rostral and caudal points of injection were offset from the midline by 0.5 mm, left and right, respectively. The wound was then sutured. After surgery, rats received daily doses of gentamicin (25 mg/kg, Omela, Russia) intramuscularly for 7 consecutive days. Bladders of injured rats were manually emptied twice daily until spontaneous voiding occurred.

### RNA isolation and real-time PCR analysis of VEGF, GDNF and EGFP

Total RNA was extracted from UCB-MCs and freshly isolated spinal cords by using Yellow Solve reagents (Silex, Russia) according to the manufacturer’s protocol. Non-transduced and transduced UCB-MCs native were analyzed 5 days after transduction. First-strand cDNA synthesis was performed using 100U Maxima Reverse Transcriptase (Thermo Scientific, USA). For cDNA synthesis, an RNA/primer/dNTP mixture containing 100 ng of RNA, 1 μl of Random hexamer primers (Litekh, Russia), and 8.5 μL of H_2_O was denatured at 65°C for 5 min and chilled to 4°C. cDNA was synthesized using 200 U of RevertAid Reverse transcriptase and 20 U of Ribolock RNase inhibitor (Thermo Scientific) in a 20-mL (<- uL?) reaction mixture. After incubation for 10 min at 25°C, the reaction was continued for another 60 min at 42°C and then terminated by heating at 70°C for 10 min.

*TaqMan real-time PCR*: cDNA were analyzed using a CFX 96 Real-Time PCR System (Bio-Rad, Hercules, CA, USA). Each PCR reaction (15 μl) contained 0.5 μl cDNA, 2.5× Reaction mixture B (Syntol, Russia), 200 nM of each primer, and the probe (100 nM) (Table 1).

**Table 1:** Primers and probes for RT-PCR.

Primer	Nucleotide sequence
VEGF-TM-Forward	ATCACCATGCAGATTATGCG
VEGF-TM-Reverse	TGCATTCACATTTGTTGTGC
VEGF-TM-Probe	[FAM]TCAAACCTCACCAAGGCCAGCA[BHQ1]
GDNF-TM-Forward	CGCTGAGCAGTGACTCAAAT
GDNF-TM-Reverse	CGATTCCGCTCTCTTCTAGG
GDNF-TM-Probe	[FAM]TCCATGACATCATCGAACTGATCAGG[BH1]
18S-TM-Forward	GCCGCTAGAGGTGAAATTCTTG
18S-TM-Reverse	CATTCTTGGCAAATGCTTTCG
18S-TM-Probe	[HEX]ACCGCGCAAGACGGACCAG[BH2]
EGFP-TM-Forward	AGCAAAGACCCCAACGAGAA
EGFP-TM-Reverse	GGCGGCGGTCACGAA
EGFP-TM-Probe	[FAM]CGCGATCACATGGTCCTGCTGG[BH1]

The following amplification parameters were used: preheating at 95°C for 3 min, followed by 39 cycles of 95°C for 10 s and 55°C for 15 s including the plate-read. mRNA was normalized to 18S ribosomal RNA. Standard curves for relative quantitation of EGFP, VEGF, and GDNF were generated using serial dilutions of plasmid DNAs containing the corresponding cDNA inserts. The expression levels after SCI were set at 100%. All RT-PCR reactions were performed in triplicate.

### Histological assessment

For histology and immunohistochemistry, 30 days after transplantation rats were anesthetized with chloral hydrate, prior to intracardiac perfusion with 4% paraformaldehyde (PFA, Sigma) as previously described [21]. After incubation in 30% sucrose, samples were frozen in liquid nitrogen and embedded in tissue freezing medium. Non-fixed tissue was used for RT-PCR and Western blot. Twenty micron longitudinal tissue sections, obtained with a Microm HM 560 Cryostat, were stained with Azur-eosin for measurement of cavity volume. The area of the cavity in the damaged spinal cord was measured in all animals. The combined area of any cysts was calculated and subtracted from the total tissue area (4.5 mm length) of intact four section to estimate total tissue remaining as described previously [22]. ImageJ Version 1.46 was used for data acquisition and analysis.

### Immunofluorescence analysis

Longitudinal tissue sections (20 μm) were incubated with primary and secondary antibodies (Abs) shown in Table 2.

**Table 2:** Primary and secondary antibodies used in immunofluorescent staining.

Antibody	Host	Dilution	Source
HNu	Mouse	1:150	Millipore
VEGF	Goat	1:300	Sigma
GDNF	Rabbit	1:100	Santa Cruz
GFAP	Mouse	1:200	Santa Cruz
CGRP	Rabbit	1:150	Santa Cruz
GAP43	Goat	1:150	Santa Cruz
Anti- goat IgG conjugated with Alexa 488	Donkey	1:200	Invitrogen
Anti- rabbit IgG conjugated with	Donkey	1:200	Invitrogen
Alexa 555			
Anti- mouse IgG conjugated with	Donkey	1:200	Invitrogen
Alexa 647			

For double and triple immunofluorescence labeling, sections were blocked with 5% normal goat serum for 1 hour at room temperature (RT) and then incubated overnight at 4°C with a mixture of primary Abs raised in distinct species. Prior to visualization, sections were incubated with fluorophore-conjugated secondary Abs for 2 h at RT. 4',6-Diamidino-2-phenylindole (DAPI) (10 μg/mL in PBS, Sigma) was used for visualizing nuclei. Coverslips were mounted on slides using mounting medium (ImmunoHistoMount, Santa Cruz). Sections were examined using an LSM 780 Confocal Microscope (Carl Zeiss, Germany). Only HNu-cells that displayed clearly outlined nuclei were evaluated. The mean intensity of labeling (semi-quantitative analysis of GAP-43) was analyzed using Zen 2012 Software (Carl Zeiss). All sections were imaged in the z-plane using identical confocal settings (laser intensity, gain, and offset). Measurements were obtained from longitudinal histological sections collected at 50-μm increments extending from the contusion center (observed area, 2 mm^2^) of the SCI or Th8 vertebral level for sham group.

### Western blotting

Total protein was isolated from spinal cords by homogenization of tissue samples in RIPA buffer (Sigma). Protein concentrations were determined using a Protein Assay BCA Kit (Thermo Scientific). Protein extracts (40 μg) were analyzed by 4%–13% gradient SDS-PAGE and then transferred to PVDF membranes. After incubation with glial fibrillary acidic protein (GFAP) (1:200, mouse monoclonal, Santa Cruz) and HRP-conjugated anti-mouse IgG (Sigma), blots were visualized with a mixture of 1,25mM luminol, 146mM p-coumaric acid and 34% H_2_O_2_. Detection and analysis of immune complexes was performed using the Gel Doc XRS+ System (Bio-Rad, Hercules, CA, USA). Bands were quantified using Image J Version 1.46, GFAP densitometric levels were normalized to β-actin. We also performed positive and negative controls using Western Blotting control for GFAP antibodies (sc-115582, Santa Cruz) and protein extracts from mononuclear umbilical cord blood cells (which does not contain GFAP), respectively.

### Behavioral assessment after SCI

Locomotor recovery was evaluated using the open-field BBB locomotor ratio scale [23]. The baseline was obtained on the three days before SCI. To evaluate differences in functional recovery, behavioral assessment in all groups was performed before SCI, on day 7, and then every day. Locomotion was scored simultaneously by 2 observers who were blinded to the treatment groups. Final scores were obtained by averaging the two scores awarded by the examiners.

### Statistical analysis

Data are presented as means ± standard error of the mean (SEM). Student’s t test, a one-way analysis of variance (ANOVA) with Tukey’s test or two-way analysis of variance (ANOVA) were used for multiple comparisons between all experimental and control groups. A value of P < 0.05 was considered statistically significant. Data were analyzed using Origin 7.0 SR0 Software (OriginLab, Northampton, MA, USA).

## Results

### Analysis of the expression of VEGF, GDNF and EGFP

**Fig 1:**
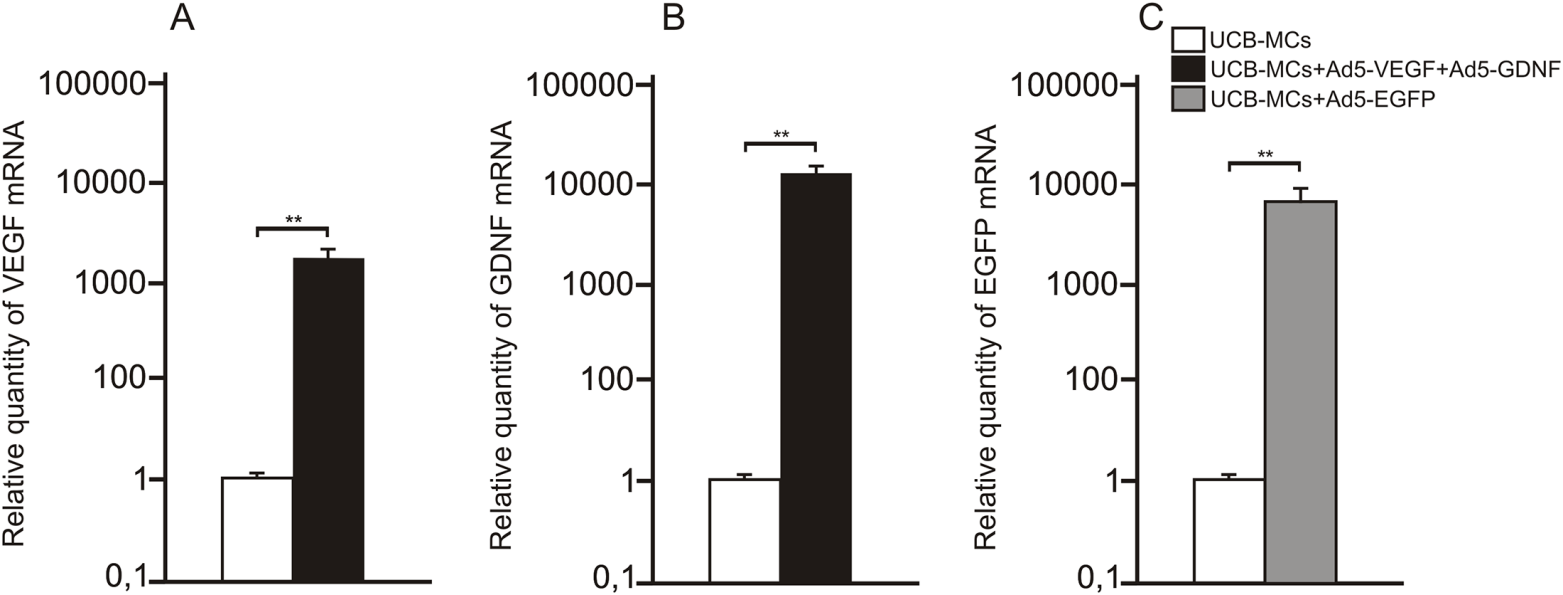
VEGF, GDNF and EGFP mRNA expression in vitro. VEGF (A), GDNF (B) and EGFP (C) mRNA expression on day 5 after transduction of UCB-MCs concurrently with adenoviral vectors Ad5-VEGF/Ad5-GDNF and Ad5-EGFP, respectively. The levels of VEGF, GDNF and EGFP mRNA expression in UCB-MCs was considered 100%. Differences were statistically significant between UCB-MCs and the experimental groups in all cases (**–P < 0.01, Student’s t-test).

Genetically modified UCB-MCs were analyzed 5 days after transduction. Expression of VEGF, GDNF and EGFP mRNA was quantified by normalizing to 18S ribosomal RNA. VEGF and GDNF mRNA levels in UCB-MCs simultaneously transduced with Ad5-VEGF and Ad5-GDNF was 2500 and 14000 times higher, respectively, relative to non-transduced UCB-MCs (Fig 1).

EGFP mRNA expression of UCB-MCs treated with Ad5-EGFP was 8500 times higher than that at this time point in non-transduced UCB-MCs. These data demonstrate robust expression levels of these transgenes in transduced UCB-MCs. The mRNA expression of transgenes was standardized against expression by non-transduced cells. Non-transduced cells expressed negligible levels of EGFP mRNA.

**Fig 2:**
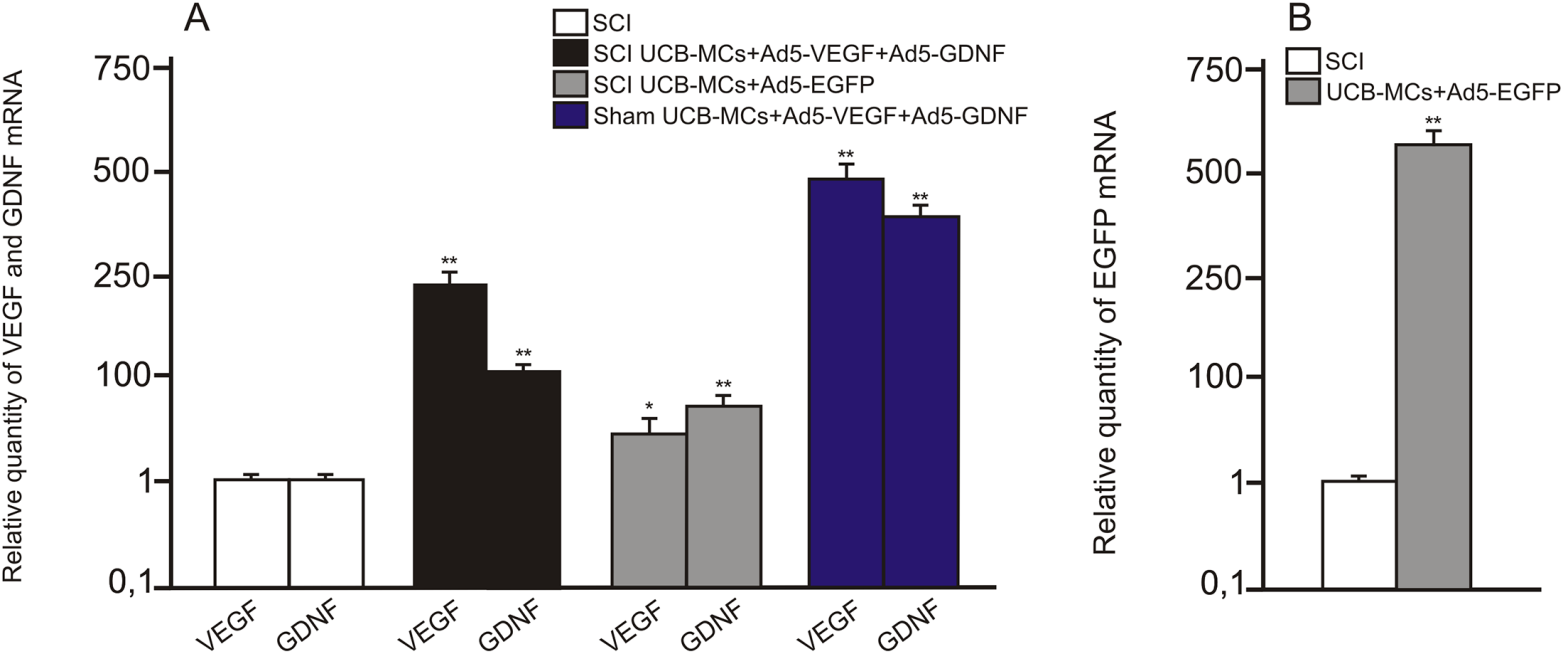
VEGF, GDNF and EGFP mRNA expression in vivo. VEGF, GDNF (A) and EGFP (B) mRNA expression on day 30 after SCI or Sham followed by injection of transduced UCB-MCs. The levels of VEGF, GDNF and EGFP mRNA expression in SCI group was considered 100%. Differences were statistically significant between SCI-group and other experimental groups (*–P < 0.05, **–P < 0.01, Student’s t-test).

To investigate whether transplantation of genetically modified UCB-MCs increased expression of VEGF, GDNF and EGFP in injured spinal cord, qRT-PCR was performed on sections obtained from the injury site 30 days post transplantation. VEGF and GDNF mRNA in the spinal cord of UCB-MCs+Ad5-VEGF+Ad5-GDNF-treated rats 30 days after injury and laminectomy was 105–245 and 492–378 times higher, respectively, than untreated rats subjected to SCI (Fig 2A). The level of VEGF and GDNF mRNA in the spinal cord of UCB-MCs+Ad5-EGFP-treated rats 30 days after injury was 5 and ~1.4 times lower, respectively, than UCB-MCs+Ad5-VEGF+Ad5-GDNF. EGFP mRNA in the spinal cord of UCB-MCs+Ad5-EGFP-treated rats on days 30 after SCI was 556 times higher than untreated rats subjected to SCI (Fig 2B). These results demonstrate that expression of VEGF, GDNF and EGFP was markedly upregulated 30 days after transplantation of genetically modified UCB-MCs.

### Measurement of the cavity volume and intact tissue

**Fig 3:**
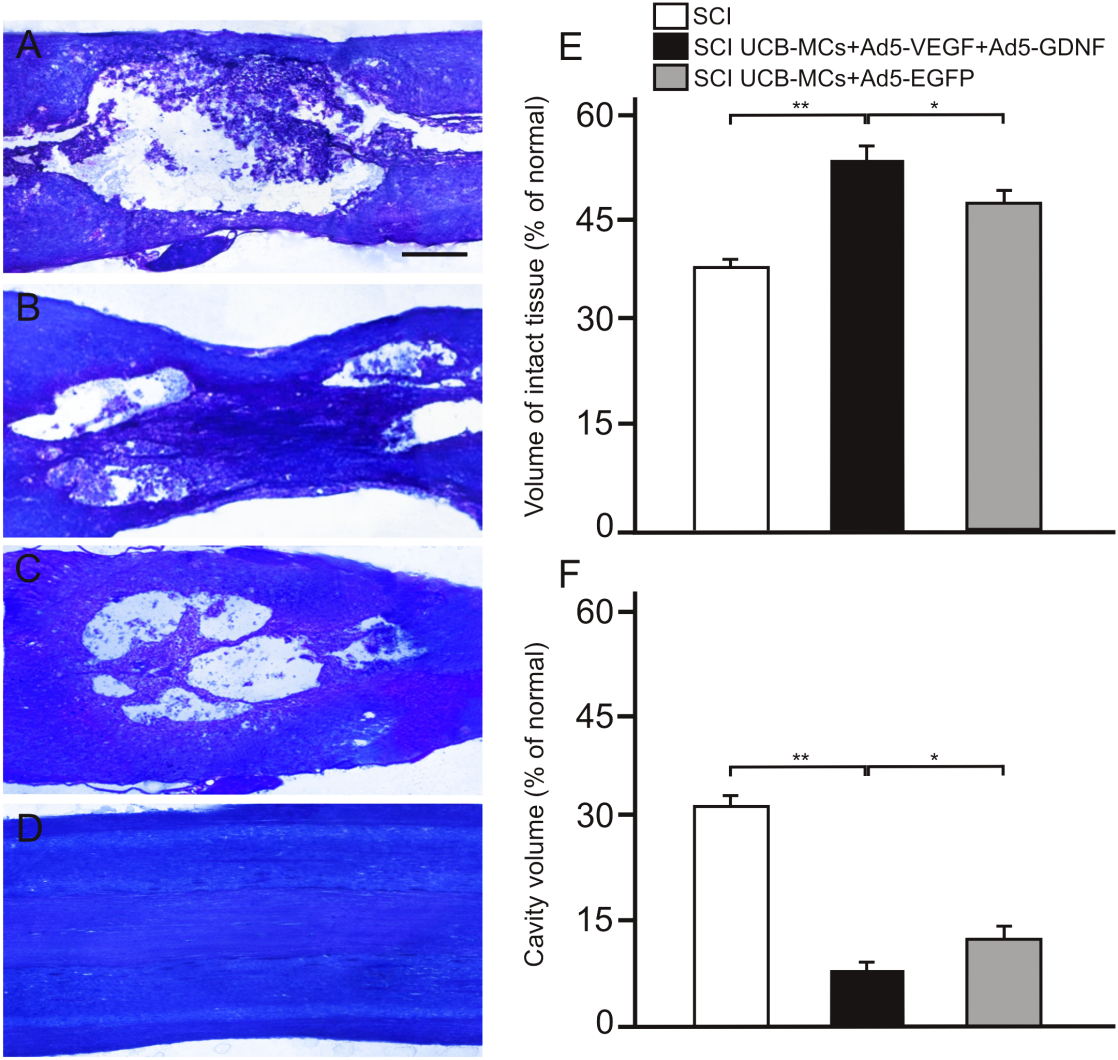
Tissue analysis in experimental groups. Injured spinal cord 30 days after SCI without therapy (A), SCI with direct injection of UCB-MCs+Ad5-VEGF+Ad5-GDNF (B) and UCB-MCs+Ad5-EGFP (C), Sham operation with direct injection of UCB-MCs+Ad5-VEGF+Ad5-GDNF (D). Images are Azur-eosin cryosections. Scale bar: 750 μm. Volume of intact tissue (E) and cavity volume (F) relative to the Th8 area of intact rats remaining 30 days after SCI in experimental groups. *–P < 0.05, **–P < 0.01, one-way ANOVA followed by a Tukey’s *post hoc* test.

Loss of tissue at the lesion site was detected in all SCI groups at day 30, consistent with our previous study of the structure of white and gray matter after SCI [10]. In the Sham UCB-MCs+Ad5-VEGF+Ad5-GDNF group (laminectomy) we did not see signs of nerve fiber degeneration (Fig 3D) although we could see minimal damage from the syringe. Whereas, a central zone of completely disintegrated tissue, containing dispersed fragments of degenerated cells, was detected in the spinal cord of SCI-only rats (Fig 3A). By comparison, in the spinal cord contusion area of UCB-MCs+Ad5-EGFP-treated rats the size of the traumatic centromedullary cavity was reduced and contained an islet of intact tissue (see below, Fig 3C). In the UCB-MCs+Ad5-VEGF+Ad5-GDNF-treated rats we detected a central zone with spared axons and a higher degree of preserved tissue, nonetheless distal and caudal parts of the injury center contained medium-sized cavities containing cell fragments and viable cells (see below, Fig 3B). At day 30 after SCI, the volume of intact tissue was 53.1±4.5% units in the UCB-MCs+Ad5-VEGF+Ad5-GDNF group, 47±1.8% units in the UCB-MCs+Ad5-EGFP group, and 38.8±1.2% units in the SCI-only group (mean±s.e.m) (Fig 3E). A significant difference was also seen between groups at day 30 in cavity volume, which was 7.4±1.3% units in the UCB-MCs+Ad5-VEGF+Ad5-GDNF group, 11.7±2.5% units in the UCB-MCs+Ad5-EGFP group and 31.9±2.1% units in the SCI-only group (Fig 3F). Thus, transplantation of UCB-MCs without a therapeutic transgene (UCB-MCs+Ad5-EGFP) reduced cavity volume and tissue retention at the lesion site. The UCB-MSCs transduced with adenoviral vectors expressing VEGF and GDNF genes led to a further reduction of cavity volume after SCI, and improvement in retention of tissue.

### Distribution and survival of transplanted UCB-MCs

**Fig 4:**
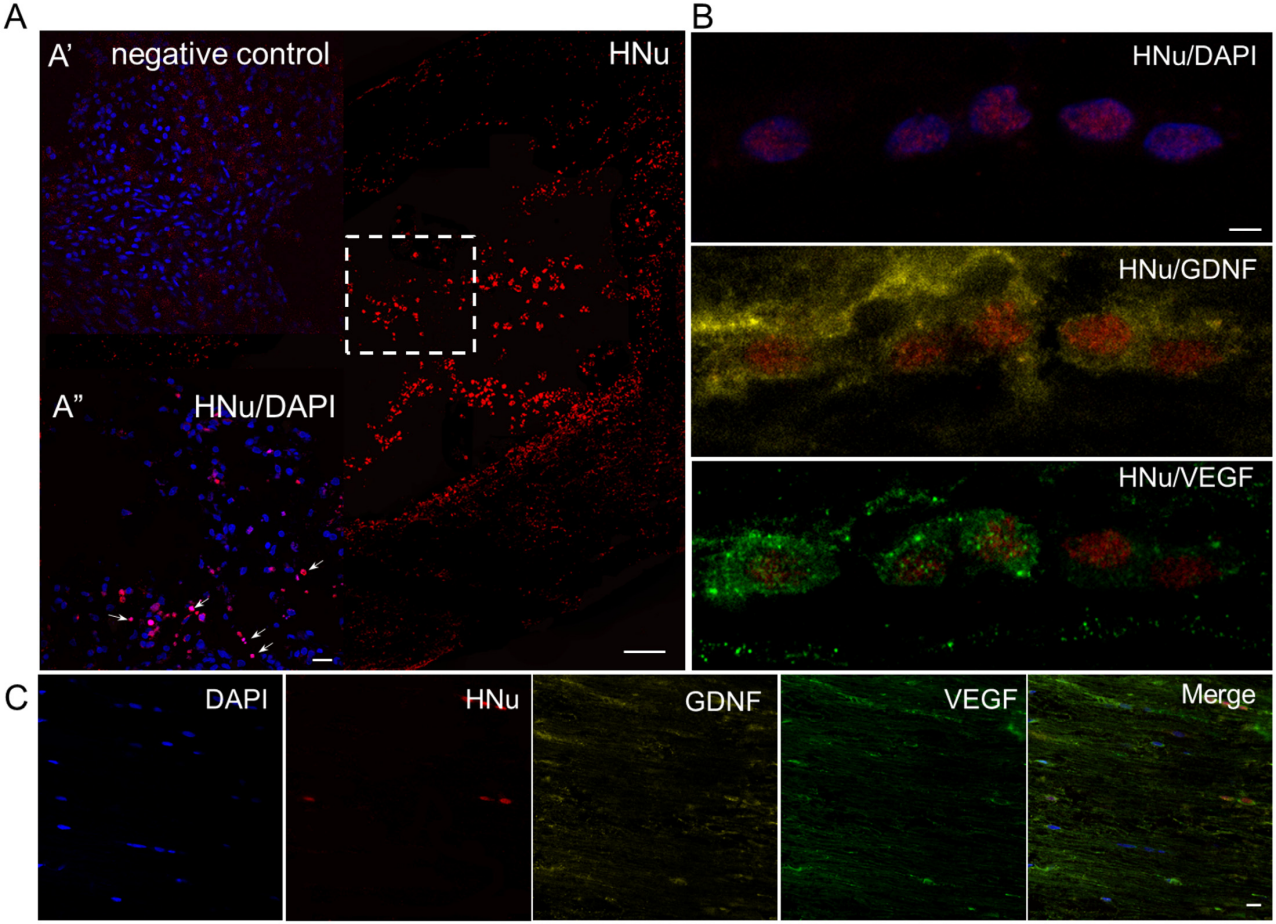
Visualization of grafted cells at day 30 after transplantation. Visualization of grafted cells in the SCI zone 30 days after transplantation of UCB-MCs+Ad5-EGFP (A) and UCB-MCs+Ad5-VEGF+Ad5-GDNF (B). Localization of transplanted UCB-MCs+Ad5-VEGF+Ad5-GDNF after Sham operation (C). (A) HNu^+^ cells (red) surviving in the spinal cord contusion area, and forming cell bridges within the traumatic centromedullary cavity. The dashed boxes indicate enlarged area of A”. Arrows show some the HNu and DAPI overlap cells which were used for analysis. (A’) The same area, labeled by dashed boxes, in subsequent serial sections was stained as negative control. (B) In UCB-MCs+Ad5-VEGF+Ad5-GDNF-treated rats the contusion area with spared tissue contained mostly HNu^+^ cells, which expressed VEGF and GDNF. (C) HNu^+^/VEGF^+^/GDNF^+^ cells arranged accordingly to the nerve fibers 30 days after Sham with injection of UCB-MCs+Ad5-VEGF+Ad5-GDNF. Nuclei are stained with DAPI (blue). Scale bar: 200 (A), 10 (A’, A”), 2,5 (B) and 20 (C) μm.

To assess the ability of transplanted human cells to survive, persist and migrate within the contusion area from the site of administration, anti-HNu Abs were used to visualize the grafted UCB-MCs in the spinal cord. Image analysis showed that UCB-MCs administered directly into the lesion site (immediately following SCI), survived 30 days after injection, consistent with detection of expression of VEGF, GDNF and EGFP. In UCB-MCs+Ad5-EGFP-treated rats HNu^+^ cells survived in the spinal cord contusion area, forming cell bridges within the traumatic centromedullary cavity (Fig 4A and 4A”). In the UCB-MCs+Ad5-VEGF+Ad5-GDNF-treated rats a few HNu^+^ cells were observed in the trauma cavities, while the contusion area with spared tissue contained mostly HNu^+^ cells (Fig 4B). Spinal cord sections triple-labeled with VEGF, GDNF and HNu Abs showed that UCB-MCs were expressing VEGF and GDNF genes in the SCI UCB-MCs+Ad5-VEGF+Ad5-GDNF and Sham UCB-MCs+Ad5-VEGF+Ad5-GDNF groups (Fig 4B and 4C). Interestingly, HNu^+^ cells arranged accordingly to the nerve fibers at 30 days after for both SCI and Sham with UCB-MCs+Ad5-VEGF+Ad5-GDNF.

### Assessment of glial scar and CGRP^+^/GAP43^+^ axonal profiles

**Fig 5:**
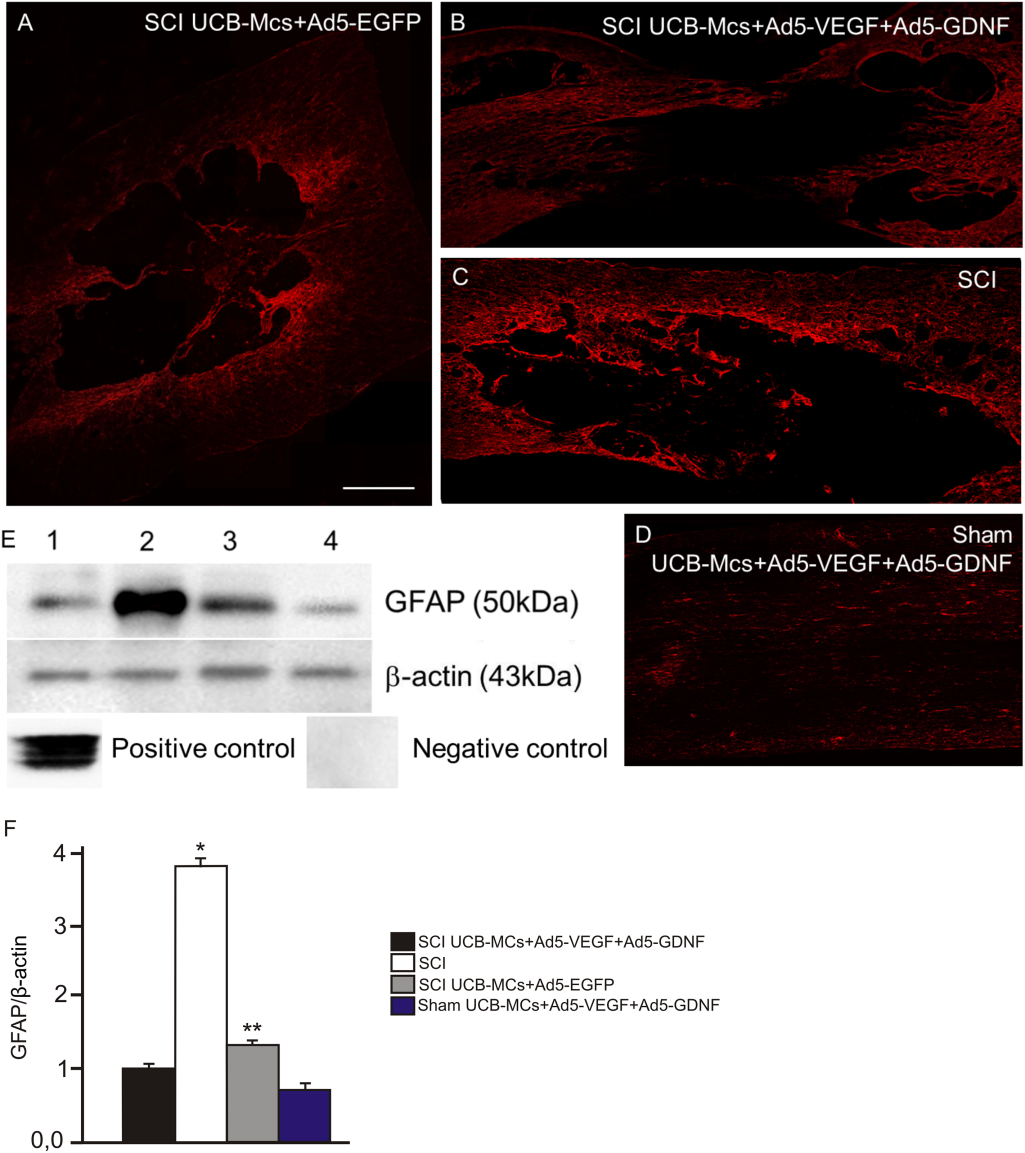
Glial scar formation at the lesion site indicated by GFAP. Visualization of glial scar formation at the lesion site using GFAP in experimental groups: SCI UCB-MCs+Ad5-EGFP (A), SCI UCB-MCs+Ad5-VEGF+Ad5-GDNF (B), SCI (C) and Sham UCB-MCs+Ad5-VEGF+Ad5-GDNF (D). In the UCB-MCs+Ad5-VEGF+Ad5-GDNF-treated rats after SCI we detected a central zone of tissue without GFAP immunofluorescence. Scale bar: 750 μm. (E) Western-blotting analysis of GFAP on the 30th day after SCI with direct injection of UCB-MCs+Ad5-VEGF+Ad5-GDNF (1) and UCB-MCs+Ad5-EGFP (3), SCI without therapy (2), Sham operation with direct injection of UCB-MCs+Ad5-VEGF+Ad5-GDNF (4). Staining with Abs against GFAP revealed a band at 50 kDa in the samples. β-actin was used as a loading control. At day 30, western blot analysis shows reduced GFAP expression at the injury zone after UCB-MCs+Ad5-VEGF+Ad5-GDNF injection. Positive and negative controls were performed using Western Blotting control for GFAP antibodies and protein extracts from mononuclear umbilical cord blood cells, respectively. (F) Densitometry analysis demonstrated a significant change in GFAP levels relative to β-actin expression after SCI. Differences were statistically significant between SCI and other experimental groups (*P < 0.01). Differences were also statistically significant between groups with injection of UCB-MCs (**P < 0.05). One-way ANOVA.

**Fig 6:**
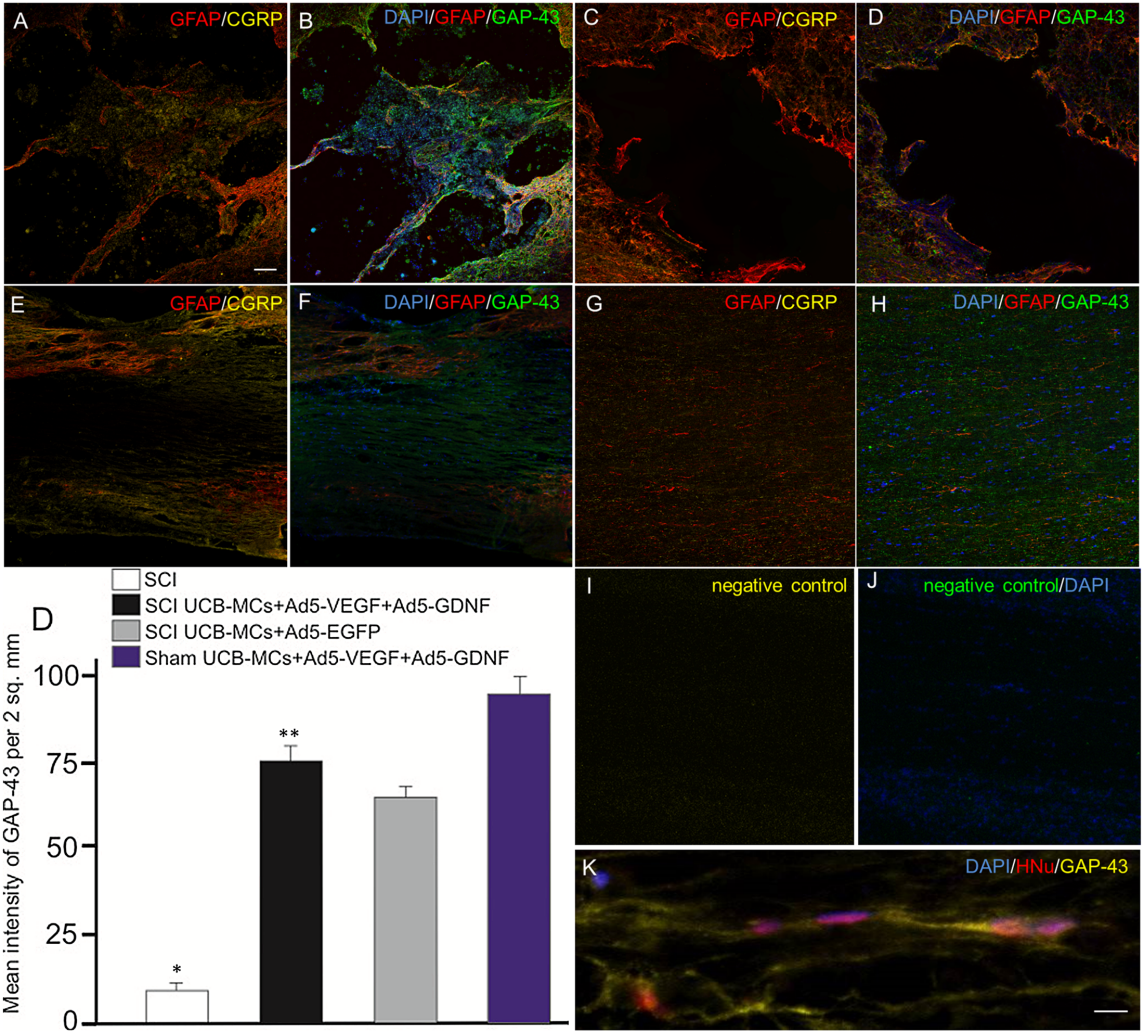
Expression of GFAP, CGRP and GAP43 in the lesion site of spinal cord. Visualization of the distribution of GFAP, CGRP and GAP43 at the site of spinal cord contusion lesion after SCI-only (C,D), transplantation of UCB-MCs+Ad5-EGFP (A,B) and UCB-MCs+Ad5-VEGF+Ad5-GDNF after SCI (E,F,K,M,N) and Sham (G,H). In the UCB-MCs+Ad5-EGFP group (A,B) CGRP and GAP-43 expression was located in the islet of lesion zone, surrounded by glial scar. The UCB-MCs at the injury site appeared to be very closely associated with GAP43^+^ axons (K). The sections I,J present negative controls. Nuclei are stained with DAPI (blue). Scale bar: 100 (A-J) and 5 (K). (D) Mean labeling intensity of GAP43 of the rats in experimental groups in the SCI center. Differences were statistically significant between SCI and other experimental groups (*P < 0.01). Differences were also statistically significant between groups with injection of UCB-MCs (**P < 0.05). One-way ANOVA.

Glial scar formation was assessed in spinal cords using immunofluorescence microscopy and western blot analysis using antibodies to GFAP. At day 30, there were lower levels of GFAP expression at the injury zone after UCB-MCs+Ad5-VEGF+Ad5-GDNF injection (Fig 5) relative to over experimental groups. The density of specific bands was determined by densitometric analysis and expressed as a ratio of β-actin. At day 30 of UCB-MCs+Ad5-VEGF+Ad5-GDNF treatment, GFAP production decreased about three-fold compared with the SCI-only group (Fig 6).

**Fig 7:**
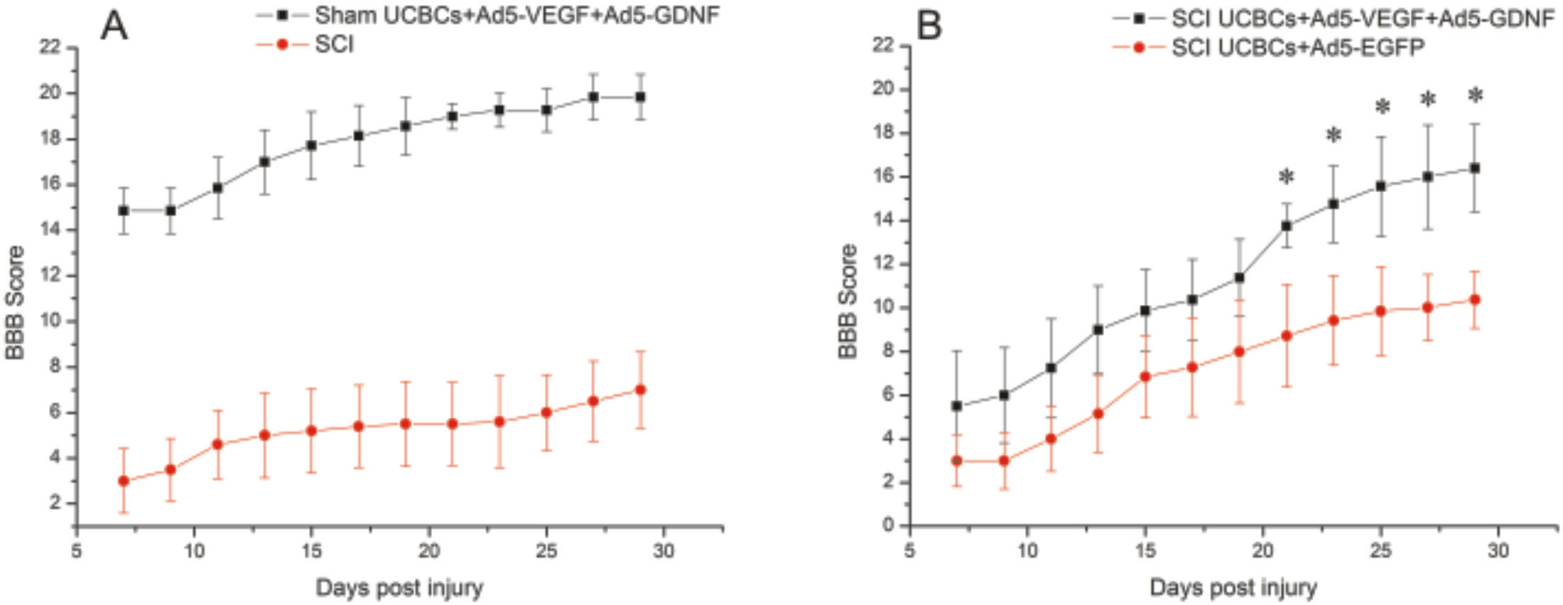
BBB locomotor scores of rats after SCI or Sham in experimental group. BBB locomotor scores of rats obtained for the SCI (A, red line), SCI UCB-MCs+Ad5-EGFP (B, red line), SCI UCB-MCs+Ad5-VEGF+Ad5-GDNF (B, black line) and Sham UCB-MCs+Ad5-VEGF+Ad5-GDNF (A, black line) groups. Statistically significant differences were detected between Sham UCB-MCs+Ad5-VEGF+Ad5-GDNF group and other groups for all days (P < 0.05). BBB scores were higher in the UCB-MCs+Ad5-EGFP (B, red line) group than in the SCI (A, red line) on at least 3 post-injury days (9, 11 and 13). *–P < 0.05, two-way ANOVA.

In UCB-MCs+Ad5-VEGF+Ad5-GDNF-treated rats after SCI we detected a central zone with no GFAP immunofluorescence (Fig 5), but with CGRP^+^ and GAP43^+^ axons (Fig 7E and 7F). The UCB-MCs at the injury site appeared to be very closely associated with GAP43^+^ axons (Fig 7K). In SCI rats at day 30, we could not detect white matter tracts of spared CGRP^+^ and GAP-43^+^ fibers in the lesion site, and there was little expression of CGRP and GAP-43 distal to the lesion zone (data not shown). Meanwhile in the EGFP group (UCB-MCs+Ad5-EGFP) CGRP and GAP-43 expression was located not only in the islet of lesion zone, but also distal to the lesion zone (Fig 7A and 7B). Furthermore, the CGRP^+^ and GAP43^+^ axonal profiles in the UCB-MCs+Ad5-VEGF+Ad5-GDNF group were clearly visible and could also be visualized in the lesion zone with no GFAP immunofluorescence.

Measurement of the mean intensity of the GAP-43 immunoreactivity in the lesion zone demonstrated that mean intensity of GAP-43 between the four groups was statistically significant (P<0.05) (Fig 7). The GAP-43 mean intensity in the SCI-only group was lower than the cell transplantation groups such as UCB-MCs+Ad5-VEGF+Ad5-GDNF and UCB-MCs+Ad5-EGFP (P<0.0001). The Sham UCB-MCs+Ad5-VEGF+Ad5-GDNF group had the highest GAP-43 mean intensity, with the mean intensity GAP-43 higher in SCI UCB-MCs+Ad5-VEGF+Ad5-GDNF group than the SCI UCB-MCs+Ad5-EGFP group. Thus, we have demonstrated that transplantation of UCB-MCs transduced with adenoviral vectors expressing VEGF and GDNF genes into the site of SCI induce prominent axonal sparing/regeneration relative to other constructs tested.

### Assessment of locomotor activity

We assessed locomotor recovery using the BBB rating scale from 7 to 30 days after injury and laminectomy. In the case of SCI-only rats at day 29 after injury, BBB scores were low (7 ± 1.67) (Fig 7A). The motor function scores of UCB-MCs+Ad5-VEGF+Ad5-GDNF-treated rats (16.14 ± 2) were higher than the UCB-MCs+Ad5-EGFP-treated rats (10.07 ± 1.3) at this time point (Fig 7B), with significant differences (P < 0.05) on at least 5 post-injury days (21, 23, 25, 27 and 29). The UCB-MCs+Ad5-VEGF+Ad5-GDNF-treated rats showed significantly higher BBB scores than did SCI controls at every time point, starting from day 13 following traumatic injury (P < 0.05). In the case of laminectomy, animals in the Sham UCB-MCs+Ad5-VEGF+Ad5-GDNF group showed an increase in the BBB score from 14.85 at day 7 to 19.85 by day 29. An average increases in the BBB scores from day 7 to 29 were 2.3, 3.85, 5.38-fold, respectively in the SCI-, SCI UCB-MCs+Ad5-EGFP- and SCI UCB-MCs+Ad5-VEGF+Ad5-GDNF-groups. Thus, the behavioral data from the BBB locomotor scores demonstrate that after SCI, UCB-MCs+Ad5-VEGF+Ad5-GDNF-treated rats had dramatically improved neurological function.

## Discussion

In this study, we demonstrated the capacity of UCB-MCs transduced concurrently with adenoviral vectors encoding VEGF and GDNF to increase tissue sparing and numbers of spared/regenerated axons, reduce glial scar formation and promote behavioral recovery when transplanted immediately after a rat contusion SCI. The adenoviral vectors encoding VEGF and GDNF, used to transduce UCB-MCs, were shown to be an effective and stable in these cells following transplantation. Viable transplanted UCB-MCs+Ad5-EGFP and UCB-MCs+Ad5-VEGF+Ad5-GDNF were found in similar numbers 30 days after SCI, but the UCB-MCs transduced with adenoviral vector encoding EGFP were more tightly distributed in the traumatic centromedullary cavity. When compared to UCB-MCs+Ad5-EGFP, the UCB-MCs transduced with VEGF and GDNF were more evenly distributed in a rostrocaudal direction, and had greater integration with spared CGRP^+^ and GAP43^+^ axons. A similar situation was observed in the Sham UCB-MCs+Ad5-VEGF+Ad5-GDNF group. Axonal staining showed tighter bundles of axons within the UCB-MCs+Ad5-VEGF+Ad5-GDNF transplant injury zone after SCI than in UCB-MCs+Ad5-EGFP and SCI (without therapy) zones. Intraspinal injection of GDNF after SCI has neuroprotective effects, supported by the expression of CGRP and GAP-43 [12]. GDNF has been demonstrated to increase the number and size of regenerating axons and to stimulate neurite outgrowth in cultures of spinal ganglion neurons [24], whereas VEGF, a typical neurotrophic factor, supports survival motor [13] and sensitive [25] neurons stimulating neurogenesis and axonal growth [14]. Therefore, we hypothesize that the combined influence VEGF and GDNF promotes axonal sparing/regeneration in the SCI UCB-MCs+Ad5-VEGF+Ad5-GDNF group by offering a growth-permissive surface.

Statistically significant tissue-protective effects of UCB-MCs transduced with adenoviral vectors encoding VEGF and GDNF was observed 30 days after transplantation. In a previous study [10], we demonstrated that injection of the UCB-MCs transduced with an adenoviral vector encoding GDNF increased tissue sparing after SCI compared to UCB-MCs+AdV-EGFP. Similarly in this study, we show that UCB-MCs+Ad5-VEGF+Ad5-GDNF increased tissue sparing relative to UCB-MCs+Ad5-EGFP. Although we did not compare UCB-MCs+Ad5-GDNF and UCB-MCs+Ad5-VEGF+Ad5-GDNF groups in this study, it would be interesting to do so in the future. The improvement in outcome after spinal injury confirms that transplanted UCB-MCs after SCI have a neuroprotective effect, however, the mechanisms of amelioration of the structural deficits remains to be elucidated.

UCB-MCs transduced concurrently with adenoviral vectors encoding VEGF and GDNF transplanted after SCI and Sham displayed significantly lower levels of GFAP immunofluorescence within and around the lesion site when compared to the UCB-MCs+AdV-EGFP and SCI-only groups. Previously, Deng et al. reported that GDNF, combined with transplanted Schwann cells, inhibits astrocyte production of the inhibitors of neurite outgrowth, GFAP and chondroitin sulfate proteoglycan, which can lead to a reduction of the inhibitory effect on the regeneration of nerve fibers by an astroglial scar [26]. There are contradictory data on the influences of VEGF on astrogliosis after injury [15, 27]. However, quantification of GFAP immunoreactivity in the present study indicated decreased astrogliosis (even in the center of the injury) after injection of UCB-MCs with VEGF and GDNF genes. We hypothesize that decreased chondroitin sulphate proteoglycans (CS56) synthesis due to reduction in glial scarring contributed to sprouting nerve fibers. Interestingly, UCB-MCs that have not been transduced with VEGF and GDNF (in this study UCB-MCs transduced with nontherapeutic gene EGFP) did not reduce glial scarring, nor did they promote functionally active sprouting axons. This confirms that transplantation of the modified umbilical cord blood cells was more effective than transplantation of unmodified UCB-MC.

Evaluation of recovery of voluntary movements by the BBB open field method is frequently used for determination of neurological status after SCI in rats. In this study, we demonstrated that both cell-mediated (UCB-MCs) EGFP and VEGF/GDNF gene delivery into site of spinal cord injury improves motor function in rats. BBB scores were consistently higher in the UCB-MCs+Ad5-VEGF+Ad5-GDNF group compared to UCB-MCs+AdV-EGFP. In our previous study [10], we demonstrated that difference between the UCB-MCs+AdV-GDNF and UCB-MCs+AdV-EGFP groups was not statistically significant at 15–29 days after injury. Using the BBB scale, many researchers have previously demonstrated a progressive recovery over time with stem cell therapy after SCI in rats [28]. However, amelioration of structural deficits in response to cell therapy was not addressed in previous studies. Our results showed that cell-mediated VEGF and GDNF gene delivery (UCB-MCs+Ad5-VEGF+Ad5-GDNF) exerted a strong positive influence on both structural and functional parameters after SCI.

## Conclusions

Genetic modification of UCB-MCs for use in transplantation after SCI is a promising strategy for enhancing posttraumatic spinal cord regeneration. Our data demonstrates that transplantation of UCB-MCs transduced with adenoviral vectors expressing VEGF and GDNF genes into the site of SCI promotes tissue sparing, behavioral recovery and axonal regeneration when compared to controls. Further histological and behavioral studies, especially at a later time points, in animals with SCI after transplantation of genetically modified UCB-MCs (overexpressing VEGF and GDNF genes) will provide additional insight into therapeutic potential of such cells.

## References

[1] Kuh SU, Cho YE, Yoon DH, Kim KN, Ha Y. Functional recovery after human umbilical cord blood cells transplantation with brain-derived neutrophic factor into the spinal cord injured rat. Acta Neurochirurgica (Wien). 2005;147(9): 985–992. 10.1007/s00701-005-0538-y 16010451

[2] Yan HB, Zhang ZM, Jin DD, Wang XJ, Lu KW. The repair of acute spinal cord injury in rats by olfactory ensheathing cells graft modified by glia cell line–derived neurotrophic factor gene in combination with the injection of monoclonal antibody IN–1. Zhonghua Wai Ke Za Zhi. 2009;47(23): 1817–1820. 20193555

[3] Kim HM, Hwang DH, Lee JE, Kim SU, Kim BG. Ex vivo VEGF delivery by neural stem cells enhances proliferation of glial progenitors, angiogenesis, and tissue sparing after spinal cord injury. PLoS One. 2009;4(3): 1–10. 10.1371/journal.pone.0004987 PMC2656622 19319198

[4] Lin WP, Chen XW, Zhang LQ, Wu CY, Huang ZD, Lin JH.Effect of neuroglobin genetically modified bone marrow mesenchymal stem cells transplantation on spinal cord injury in rabbits. PLoS One. 2013;8(5): 1–9. 10.1371/journal.pone.0063444 PMC3642116 23658829

[5] Jones LL, Oudega M, Bunge MB, Tuszynski MH. Neurotrophic factors, cellular bridges and gene therapy for spinal cord injury. J Physiol. 2001;533(1): 83–89. 11351016 10.1111/j.1469-7793.2001.0083b.x PMC2278599

[6] Gluckman E, Locatelli F. Umbilical cord blood transplants. Opin Hematol. 2000;7(6): 353–357. 10.1097/00062752-200011000-00006 11055508

[7] Weiss M, Troyer D. Stem cells in the umbilical cord. Stem Cell Rev. 2007;2: 155–162. 10.1007/s12015-006-0022-y PMC3753204 17237554

[8] Park SI, Lim JY, Jeong CH, Kim SM, Jun JA, Jeun SS, et al Human umbilical cord blood-derived mesenchymal stem cell therapy promotes functional recovery of contused rat spinal cord through enhancement of endogenous cell proliferation and oligogenesis. J Biomed Biotechnol. 2012: 1–8. 22500090 10.1155/2012/362473 PMC3304690

[9] Rodrigues LP, Iglesias D, Nicola FC, Steffens D, Valentim L, Witczak A, et al Transplantation of mononuclear cells from human umbilical cord blood promotes functional recovery after traumatic spinal cord injury in Wistar rats. Braz J Med Biol Res. 2012;45(1): 49–57. 22183246 10.1590/S0100-879X2011007500162 PMC3854143

[10] Mukhamedshina YO, Shaymardanova GF, Garanina EE, Salafutdinov II, Rizvanov AA, Islamov RR, et al Adenoviral vector carrying glial cell-derived neurotrophic factor for direct gene therapy or human umbilical cord blood cell-mediated therapy of spinal cord injury in rat. Spinal Cord. 2015 9 29 10.1038/sc.2015.161 [Epub ahead of print] 26415641

[11] Ikeda YN, Fukuda M, Wada T, Matsumoto A, Satomi S, Yokoyama S, et al Development of angiogenic cell and gene therapy by transplantation of umbilical cord blood with vascular endothelial growth factor gene. Hypertens Res. 2004;27(2): 119–128. 15005275 10.1291/hypres.27.119

[12] Cheng H, Wu JP, Tzeng SF. Neuroprotection of glial cell line–derived neurotrophic factor in damaged spinal cords following contusive injury. Neurosci Res. 2002;69(3): 397–405. 10.1002/jnr.10303 12125080

[13] Islamov RR, Chintalgattu V, Pak ES. Induction of VEGF and its Flt-1 receptor after sciatic nerve crush injury. Neuroreport. 2004;15(13): 2117–2121. 15486493 10.1097/00001756-200409150-00024

[14] Mackenzie F, MacRuhrberg C. Diverse roles for VEGF–A in the nervous system. Development. 2012;139(8): 1371–1380. 10.1242/dev.072348 22434866

[15] Rosenstein JM, Mani N, Silverman WF, Krum JM. Patterns of brain angiogenesis after vascular endothelial growth factor administration in vitro and in vivo. Proc Natl Acad Sci USA. 1998;95(12): 7086–7091. 9618543 10.1073/pnas.95.12.7086 PMC22748

[16] Jin H, Liu ML, Kim HA, Lee M, An S, Oh J, et al Role of the oxygen–dependent degradation domain in a hypoxia–inducible gene expression system in vascular endothelial growth factor gene therapy. Spine. 2009;34(26): 952–958. 10.1097/BRS.0b013e3181c4af80 20010384

[17] Sondell M, Sundler F, Kanje M. Vascular endothelial growth factor is a neurotrophic factor which stimulates axonal outgrowth through the flk–1 receptor. Eur J Neurosci. 2004;12: 4243–4254. 10.1046/j.0953-816x.2000.01326.x 11122336

[18] Mills CD, Allchorne AJ, Griffin RS, Woolf CJ, Costigan M. GDNF selectively promotes regeneration of injury–primed sensory neurons in the lesioned spinal cord. Mol Cell Neurosci.2007;36(2): 185–194. 17702601 10.1016/j.mcn.2007.06.011 PMC2034440

[19] Islamov RR, Rizvanov AA, Mukhamedyarov MA, Salafutdinov II, Garanina EE, Fedotova VY, et al Symptomatic Improvement, Increased Life-Span and Sustained Cell Homing in Amyotrophic Lateral Sclerosis After Transplantation of Human Umbilical Cord Blood Cells Genetically Modified with Adeno-Viral Vectors Expressing a Neuro-Protective Factor and a Neural Cell Adhesion Molecule. Curr Gene Ther. 2015;15(3): 266–276. 25619885 10.2174/1566523215666150126122317

[20] Lebedev SV, Timofeyev SV, Zharkov AV, Schipilov VG, Chelyshev JA, Masgutova GA, et al Exercise tests and BBB method for evaluation of motor disorders in rats after contusion spinal injury. Bull Exp Biol Med. 2008;146(4): 489–494. 19489327 10.1007/s10517-009-0328-2

[21] Mukhamedshina YO, Shaymardanova GF, Muhitov AR, Salafutdinov II, Rizvanov AA, Zarubina VN, et al Survival and differentiation of endogenous Schwann cells migrating into spinal cord under the influence of neurotrophic factors. Cellular Transplantation and Tissue Engineering. 2012;7(3): 125–129.

[22] Barbour HR, Plant CD, Harvey AR, Plant GW. Tissue sparing, behavioral recovery, supraspinal axonal sparing/regeneration following sub-acute glial transplantation in a model of spinal cord contusion. BMC Neurosci. 2013 9 27;14:106 10.1186/1471-2202-14-106 24070030 PMC3849889

[23] Basso DM, Beattie MS, Bresnahan JC. A sensitive and reliable locomotor rating scale for open field testing in rats. J Neurotrauma. 1995;12(1): 1–21. 7783230 10.1089/neu.1995.12.1

[24] Zhang L, Ma Z, Smith GM, Wen X, Pressman Y, et al GDNF-enhanced axonal regeneration and myelination following spinal cord injury is mediated by primary effects on neurons. Glia. 2009;57(11): 1178–1191. 10.1002/glia.20840 19170182 PMC2855953

[25] Sondell M, Lundborg G, Kanje M. Vascular endothelial growth factor has neurotrophic activity and stimulates axonal outgrowth, enhancing cell survival and Schwann cell proliferation in the peripheral nervous system. Neurosci. 1999;19(14): 5731–5740. 10.1523/JNEUROSCI.19-14-05731.1999 PMC6783109 10407014

[26] Deng LX, Hu J, Liu N, Wang X, Smith GM, Wen X, et al GDNF modifies reactive astrogliosis allowing robust axonal regeneration through Schwann cell-seeded guidance channels after spinal cord injury. Exp Neurol. 2011;229(2): 238–250. 10.1016/j.expneurol.2011.02.001 21316362 PMC3100372

[27] Lutton C, Young YW, Williams R, Meedeniya AC, Mackay-Sim A, Goss B. Combined VEGF and PDGF treatment reduces secondary degeneration after spinal cord injury. J Neurotrauma. 2012;29(5): 957–970. 10.1089/neu.2010.1423 21568693

[28] Antonic A, Sena ES, Lees JS, Wills TE, Skeers P, Batchelor PE, et al Stem cell transplantation in traumatic spinal cord injury: a systematic review and meta-analysis of animal studies. PLoS Biol. 2013;11(12): 1–14. 10.1371/journal.pbio.1001738 PMC3866091 24358022

